# Health Problems and Disabilities Among the Postmenopausal Saudi Women in Bisha City Receiving Home Care: A Descriptive Cross-Sectional Study

**DOI:** 10.7759/cureus.55307

**Published:** 2024-03-01

**Authors:** Hassan Alshamrani, Elhadi Miskeen, Abdullah A Alshomrany

**Affiliations:** 1 College of Medicine, University of Bisha, Bisha, SAU; 2 Department of Obstetrics and Gynecology, College of Medicine, University of Bisha, Bisha, SAU

**Keywords:** health problems and disabilities, saudi arabia, bisha, women, postmenopausal

## Abstract

Background: Postmenopausal women experience physical and psychological changes that may affect their health status. In Saudi Arabia, where the population of postmenopausal women is increasing, there is a need to examine the health problems and disabilities experienced by this group, particularly those who receive home care. This study aims to identify the common health problems and disabilities experienced by postmenopausal Saudi women in Bisha city who receive home care services.

Methods: A cross-sectional study in Bisha city, Saudi Arabia, involved 155 postmenopausal women (age 60 years and above) receiving home care services. Data were collected using structured interviews and medical records.

Results: The study found that the most common health problems among postmenopausal women receiving home care were cardiovascular diseases in 85 women (54.84%), diabetes in 85 women (54.84%), and musculoskeletal disorders in 56 women (36.13%). There was a significant association between the number of health problems and disabilities, indicating that women with more health problems were more likely to experience disabilities (p-value ≤ 0.05). The results showed that age (OR=1.56, 95% CI 1.23-1.99, p=0.001), chronic diseases (OR=2.34, 95% CI 1.43-3.84, p=0.001), and lower education level (OR=1.45, 95% CI 1.01-2.08, p=0.045) were significantly associated with the presence of health problems and disabilities among postmenopausal Saudi women receiving home care in Bisha city. However, marital status and employment status were not found to be significant predictors.

Conclusion: Postmenopausal women in Bisha city who receive home care services experience a range of health problems and disabilities, particularly related to hypertension, diabetes, and musculoskeletal disorders. The findings of this study can help healthcare providers develop effective interventions and strategies to improve the health outcomes of this population.

## Introduction

Health in middle-aged and older women demands unique attention globally due to the adversity of indisposition that affects women in their logical aging procedure. In women, procreant viability can be grouped into three phases elaborated by the hormonal swaps and periodicity of the menstrual sequence; these phases are perimenopause, menopause, and postmenopause [1]. Menopause is indicated or said to be onset, when a woman fails to menstruate for one year or consecutive months, without any health problems associated with psychological and pathological inducement [2]. Regarding the duration of menopause, its symptoms, and its onset age, various studies have shown disparities. According to a recent overview, the average menopausal period is estimated to be from five to ten years, with a standard age of 51 years for the stage onset [3].

Postmenopause is the stage that follows menopause; it is the phase after the irreparable termination of the menstrual cycle by the body's hormones for more than a year. During this phase, women tend to develop several chronic disorders that lead to disabilities and can hinder their quality of life. Such disorders include diabetes and cardiovascular diseases among others [4]. Menopausal symptoms intensify throughout the postmenopausal period compared to other stages of the menopause. The incidence of postmenopausal symptoms is significantly influenced by a number of underlying factors such as lifestyle, physiological, and cultural factors. This has been witnessed in different women and regions around the globe; for instance in KSA, a study on this phenomenon indicated that most recurring menopausal symptoms among women were discomfort of the heart, sleeping disorders, and adverse joint problems among other manifestations of the body, as women progress to the postmenopausal stage [3].

Cardiovascular risk factors such as chest discomfort or pain are some of the health problems in postmenopausal women due to hormonal changes that occur in their body like the complete loss of estrogen. This discomfort of the heart has become of interest as previous studies have depicted that more than 50% of postmenopausal women are likely to acquire hypertension [4]. Most of the diseases that occur in elderly women are linked to menopause experiences [5]. Women have a life expectancy of 6-8 years more than men. Hence, they are likely to experience most of the health problems that come with old age and they include chronic disorders [6]. The same cohort of women also have a greater susceptibility to disabilities and diseases such as back and joint pains and osteoporosis is very common [7]. This research will be beneficial for the postmenopausal population, other researchers in the future, home care unit workers, and primary health care workers. Understanding the health problems such as chronic diseases and disabilities affecting this vulnerable group with ensure easy treatment and management of their condition. Additionally, there is scanty research on the topic and this study will add great value to the existing information. This research aimed to identify the common health problems and disabilities experienced by postmenopausal Saudi women in Bisha city who receive home care services.

## Materials and methods

Study design

A descriptive cross-sectional study was carried out among 155 postmenopausal women (age 60 years and above) in elderly home care facilities in Bisha City, Saudi Arabia from January 2020 to November 2021. To determine the appropriate research design for the study, we used a cross-sectional study design, which examined the health problems and disabilities among postmenopausal Saudi women receiving home care at a specific point in time.

Study area

The study was conducted in Bisha province southwest Saudi Arabia. Bisha is in the northern part of the Asir region, about 2,000 feet above sea level. Bisha has an estimated population of 398,256 according to the 2018 Census. Approximately 240 villages extend out on both sides of the Bisha Valley, the longest valley in the country, and there are about 58 urban centers for gatherings.

Study population

The study was conducted on postmenopausal Saudi women in Bisha province who were visited by elderly home care facilities in Bisha city, Saudi Arabia.

Inclusion Population

Postmenopausal women above the age of 60 who received elderly home care facilities and women residing within Bisha who were in the postmenopausal stage were included.

Exclusion Population

Female adults within their menopause age, women with primary ovarian insufficiency, and postmenopausal women residing outside Bisha city were excluded.

Sampling

We defined our target population as postmenopausal Saudi women in Bisha City receiving home care. When selecting participants, factors such as age, socioeconomic status, and medical conditions were considered.

Plan of data collection

After obtaining permission from elderly home care facilities in Bisha city, Saudi Arabia, we conducted our study. The data included demographic variables and chronic diseases among the respondents obtained using questionnaires developed by Jasien, Izumi, and AlDughaither [7-9]. We identified the variables to be measured, such as specific health problems and disabilities, and developed a data collection tool or questionnaire. We ensured the tool is culturally sensitive and appropriate for the target population. We have considered using validated instruments or adapting existing tools with permission and trained data collectors to ensure consistency in data collection.

Data analysis

An impartial biostatistician put the replies into Microsoft Excel and ran statistical analyses on them. The necessary statistical analysis was performed using IBM SPSS Statistics for Windows, Version 23 (Released 2015; IBM Corp., Armonk, New York, United States). Frequencies and percentages were used to present continuous variables. The Pearson Chi-square test was used to examine the relationship between categorical variables. Statistical significance was defined as a probability value (p-value) less than 0.05. Logistic regression was done to predict the factors. Inferential statistics, such as chi-square tests or logistic regression, were employed to examine associations between variables or risk factors.

Ethical consideration

Approval was obtained from the ethical search committee at Bisha College of Medicine and elderly home care facilities in Bisha city, Saudi Arabia. We ensured participant confidentiality and obtained informed consent, and participation was voluntary. We addressed any potential risks or harm to participants and implemented measures to mitigate them.

## Results

The respondents who participated in this study were 155. All of them were postmenopausal Saudi women with health problems and disabilities. Most of the participants were aged between 71 and 80 years (30.97%) while the least represented age group was above 101 years (5.81%). Regarding marital status, the vast majority were divorced (53.55%) while 51 (32.90%) were widowed and only 21 (13.55%) were married. The vast majority were illiterate 126 (81.29%) and only 3 (1.94%) had attained a university level of education. As far as body mass index (BMI) is concerned, the majority of women had normal weight (42.58%) while a significantly large proportion was overweight (38.06%), and about 26 (16.78%) were obese. The majority of the respondents had four to seven children (44.52%) while the least proportion of them had between zero and two children (9.03) as shown in Table 1.

**Table 1:** Table representing the demographic variables of the respondents

Variable	Category	Frequency	Percentage
Age Group	61-70	24	15.48%
	71-80	48	30.97%
	81-90	40	25.81%
	91-100	34	21.94%
	Above 101	9	5.81%
Marital Status	Divorced	83	53.55%
	Married	21	13.55%
	Widow	51	32.90%
Education Level	Illiterate	126	81.29%
	Primary	17	10.97%
	Secondary	9	5.81%
	University	3	1.94%
Body Mass Index (BMI)	Below 18.5	4	2.58%
	18.5-24.9	66	42.58%
	25.0-29.9	59	38.06%
	30.0-35.0	23	14.84%
	Above 35	3	1.94%
Number of Children	Zero to Two	14	9.03%
	Three to Four	16	10.32%
	Four to Seven	69	44.52%
	Eight or more	56	36.13%

**Figure 1:**
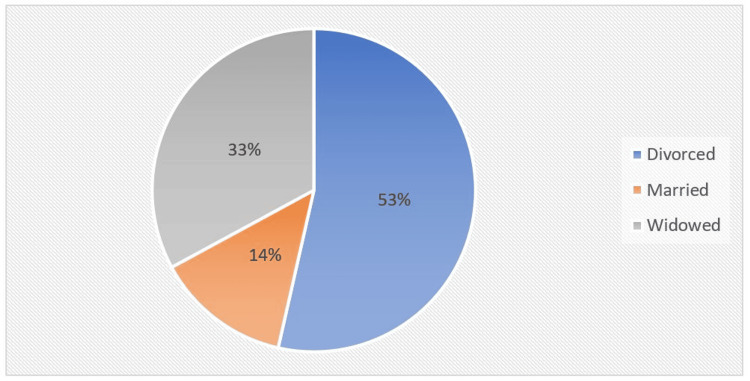
Pie chart representing the marital status of respondents

Regarding the marital status of the respondents. The pie chart visually implies that more than half (53%) of the respondents were divorced and only 14% were married. The rest (33%) were widowed as shown in Figure 1.

**Figure 2:**
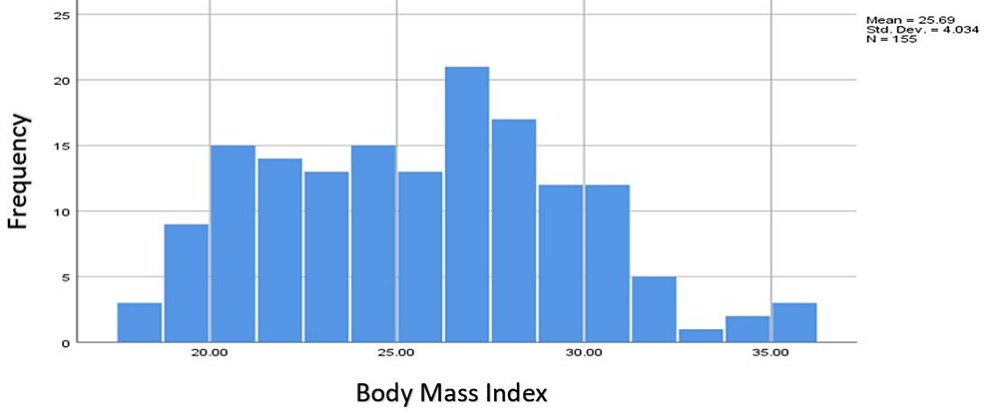
Histogram representing the BMI of the respondents

The histogram below shows the BMI distribution of the respondents. We observed a near-normal distribution with the vast majority having either normal BMI or being overweight. Just a handful of respondents were underweight or obese as shown in Figure 2.

Chronic diseases among the respondents

Multiple-choice questions on the chronic diseases affecting the respondents were posted. It was observed that cardiovascular diseases and diabetes were the most prevalent (54.84% each); dyslipidemia (33.55%), and joint and back pain (36.13%) were significantly common too. Kidney diseases, liver and gallbladder diseases, osteoporosis, visual impairment, and respiratory diseases were also common. The least prevalent chronic disease was cancer with only three incidences as shown in Table 2.

**Table 2:** Chronic diseases among the respondents

Chronic Disease	Frequency	Percentage
Cancer	3	1.94%
Cardiovascular Diseases	85	54.84%
Diabetes Mellitus	85	54.84%
Dyslipidemia	52	33.55%
Joint and Back Pain	56	36.13%
Kidney Disease	43	27.74%
Liver and Gallbladder Diseases	28	18.06%
Osteoporosis	47	30.32%
Psychological Health-Related Factors	44	28.39%
Respiratory Diseases	45	29.03%
Visual and Mode Disturbances	37	23.87%

As seen in Table 3, there was statistical significance between the age of respondents and the prevalence of chronic diseases. The associated p-value was 0.001< 0.05 which implies that age affects the prevalence. Additionally, the BMI index also showed statistical significance, and the p-value was 0.002<0.05. The education level also was a predictor for chronic diseases. It is hence imperative to note that BMI, age, and education level of respondents influenced susceptibility to chronic diseases. Other variables like marital status and number of children did not show any statistical significance as the p-values were greater than the predetermined p-value of 0.05.

**Table 3:** Association between demographic variables and prevalence of chronic diseases

Variable	Category	Frequency	Percentage	p-value
Age group	61-70	24	15.48%	0.001
	71-80	48	30.97%	
	81-90	40	25.81%	
	91-100	34	21.94%	
	Above 101	9	5.81%	
Marital Status	Divorced	83	53.55%	0.567
	Married	21	13.55%	
	Widow	51	32.90%	
Education Level	Illiterate	126	81.29%	0.016
	Primary	14	9.03%	
	Secondary	10	6.45%	
	University	5	3.23%	
Body Mass Index (BMI)	Below 18.5	4	2.58%	0.002
	18.5-24.9	66	42.58%	
	25.0-29.9	59	38.06%	
	30.0-35.0	23	14.84%	
	Above 35	3	1.94%	
Number of Children	Zero to Two	14	9.03%	0.056
	Three to Four	16	10.32%	
	Four to Seven	69	44.52%	
	Eight or more	56	36.13%	

The vast majority of respondents claimed that their menstrual cycle stopped spontaneously by a normal process (88.39%) and a small fraction (11.61%) said it was through surgical ovary removal. When the question on smoking behavior was posed, none of the respondents was a smoker. Also, none of the respondents used menopausal hormone therapy. Regarding consumption of caffeine, the vast majority of respondents did not consume caffeine (54.19%) while a fairly huge proportion (45.81%) consumed caffeine as shown in Table 4. 

**Table 4:** Results of other important research questions in the study

Research Question	Response	Frequency	Percentage
Why Menstrual Cycle Stopped	Spontaneous Natural	137	88.39%
	Surgical Ovary Removal	18	11.61%
Do You Smoke	Yes	0	0.00%
	No	155	100.00%
Use of Menopausal Hormone Therapy	Yes	0	0.00%
	No	155	100.00%
Do You Consume Drinks With Caffeine?	Yes	71	45.81%
	No	84	54.19%
How Many Meals Do You Eat Per Day?	1	15	9.68%
	2	25	16.13%
	3	54	34.84%
	More Than 3	61	39.35%

Health problems and disabilities among postmenopausal and their prediction by logistic regression

The results showed that age (OR=1.56, 95% CI 1.23-1.99, p=0.001), chronic diseases (OR=2.34, 95% CI 1.43-3.84, p=0.001), and lower education level (OR=1.45, 95% CI 1.01-2.08, p=0.045) were significantly associated with the presence of health problems and disabilities among postmenopausal Saudi women receiving home care in Bisha city. However, marital status and employment status were not found to be significant predictors as shown in Table 5.

**Table 5:** Health problems and disabilities among postmenopausal women and their prediction by logistic regression

Health Problems and Disabilities Among Postmenopausal Women	Odd ratio (OR), 95% Confidence interval (CI)
Age	OR=1.56, 95% CI 1.23-1.99, p=0.001
Chronic Diseases	OR=2.34, 95% CI 1.43-3.84, p=0.001
Lower Education Level	OR=1.45, 95% CI 1.01-2.08, p=0.045

## Discussion

The objective of this study is to determine the health problems and disabilities among postmenopausal Saudi women in Bisha province and to find out the association between demographic variables like age on chronic disease prevalence. Postmenopausal age is a critical concern for women’s health. Early menopause and late menopause have been associated with many chronic diseases and complications [10]. The results from our study indicate that age, education level, and BMI were associated with postmenopausal health problems. These findings are consistent with a study conducted in Japan which indicated that both age and BMI are critical predictors of postmenopausal health problems [8].

A study conducted by Abdel-Salam et al. in Saudi Arabia indicated that postmenopausal health problems were significantly affected by age and lifestyle behaviors such as smoking and caffeine consumption [11]. These findings are consistent with our findings that assert that dietary behavior, as well as age, affects postmenopausal health problems and more chronic diseases. In this study, the most prevalent chronic diseases for women in post-menopause age are cardiovascular diseases and diabetes both with 54.84%. Other common chronic diseases included joint and back pain (36.13%), visual and mode disturbances (23.87%), and osteoporosis (30.32%). In a study conducted by Gartlehner and colleagues on the prevention of chronic related diseases among postmenopausal women, diabetes, heart diseases, and osteoporosis were the most common chronic diseases [12]. A similar study in Riyadh, Saudi Arabia affirmed CVD and diabetes to be the most prevalent chronic disease among the elderly [9].

The BMI is an important predictor of chronic illness [13]. The vast majority of our respondents were either overweight or obese and this poses a bigger risk to the susceptibility of chronic diseases. There was a statistical association between BMI and chronic disease (P value =0.002). A study conducted by van Uffelen and colleagues on the effect of BMI on women in their seventies showed that women who had a BMI above 28 were more prone to chronic diseases such as diabetes, hypertension, and osteoporosis. However, the study alluded that other risk factors such as exercise and diet played a big role [14].

Dietary behavior among postmenopausal women is a serious matter as far as chronic-related diseases are concerned [15]. The vast majority of our respondents consumed three or more meals a day (74.19%) but the quality of their diet was not evaluated. Previous studies allude that postmenopausal people should have at least three meals a day and consume a whole-food diet with high-protein foods and phytoestrogens that are essential for improved health [16].

The use of menopausal hormone therapy is very imperative in reducing health problems such as cardiovascular disease in postmenopausal persons [17]. Although none of the participants in our study had utilized it, research has shown how important it is. In a research study by Turgeon and associates, women over 50 who had menopausal hormone therapy had a markedly lower incidence of cardiovascular illnesses [18,19]. However, there was not sufficient justification for how the therapy works but some respondents claimed that it reduces hot flashes, especially in women below the age of 60 years.

Limitations

The success of this study was flawed by several factors including difficulty in accessing respondents from other non-medical faculties. In addition, this study sought to seek and acquire sensitive personal data from elderly respondents most of whom were illiterate hence the difficulty in data collection. Cultural factors were also at play as female respondents are always reluctant to share personal medical information based on the fear of stigmatization by others. Lack of cooperation was also observed with some respondents, some of whom eventually agreed to cooperate while others opted out of the process. Another limitation not mentioned is the discrepancy in the age women hit menopause. Women who hit menopause earlier are more likely to suffer profound disabilities compared to those who had it later.

There is only a handful of prior research on postmenopausal women. A literature review was limited to a few studies. In addition to few sources for the literature review, the methodology was a limitation this took a lot of time to come up with proper research methods to conduct this study.

Implications of the research for healthcare

By implementing these implications, healthcare providers and policymakers can work toward improving the health outcomes and quality of life for postmenopausal Saudi women receiving home care in Bisha City. It is important to tailor interventions and strategies to the specific needs and cultural context of the population, fostering a holistic and patient-centered approach to healthcare delivery.

Potential Implications

Increased awareness and education: The study findings should prompt healthcare providers to raise awareness about the specific health problems and disabilities prevalent among postmenopausal Saudi women in Bisha city. Education programs can be developed to inform women, their families, and the community about the risks, symptoms, and available support services related to these health issues.

Tailored healthcare services: The study underscores the importance of designing healthcare services that are specifically tailored to meet the unique needs of postmenopausal Saudi women receiving home care in Bisha city. Healthcare providers should strive to develop patient-centered care plans that address the identified health problems and disabilities, considering the cultural and social context of the population.

Access to specialist care: The study may highlight the need for improved access to specialized healthcare services for postmenopausal Saudi women in Bisha city. Efforts should be made to enhance the availability of healthcare professionals with expertise in menopause-related health issues, chronic conditions, and disabilities to ensure timely diagnosis, treatment, and management.

Collaborative care approach: The findings emphasize the importance of adopting a multidisciplinary and collaborative care approach. Healthcare providers, including primary care physicians, specialists, nurses, and allied healthcare professionals, should work together to develop comprehensive care plans that address the physical, emotional, and social aspects of the identified health problems and disabilities.

Home care services enhancement: The study findings may prompt healthcare policymakers and administrators to assess and enhance home care services available to postmenopausal Saudi women in Bisha city. This may involve expanding the scope of home care services to include comprehensive assessments, specialized care, rehabilitation programs, and support for activities of daily living to improve the overall quality of life for these women.

Policy and guidelines development: The study's implications can contribute to the development of policies and guidelines specific to the healthcare needs of postmenopausal Saudi women receiving home care in Bisha city. This can help standardize care practices, establish quality assurance mechanisms, and ensure equitable access to healthcare services for this population.

Research and evaluation: The study findings should encourage further research to explore the underlying causes, risk factors, and long-term consequences of the identified health problems and disabilities. Longitudinal studies, qualitative research, and interventions can provide valuable insights into effective strategies for prevention, early intervention, and management of these health issues.

Recommendations

There are just a handful of studies on the health problems among postmenopausal women diseases, especially in Saudi Arabia. This calls for in-depth research on the same. Also, there is a significantly low level of awareness about postmenopausal risk factors among the women involved in this study; more awareness or education can be conducted on the same. We recommend more vigorous awareness campaigns coupled with free testing of chronic diseases, especially among elderly women so that more women are aware of their health status and know how to manage their situations. The stakeholders from different sectors like the Ministry of Health can be involved in mass screening and awareness campaigns.

## Conclusions

In our current study, postmenopausal women complain about a variety of health issues, particularly chronic disorders. Age, BMI, and level of education were very important predictors of health problems. Additionally, dietary behaviors and the use of menopausal hormone therapy were other important predictors. Addressing postmenopausal signs among aging women is critical, and health practitioners must be very careful about this vital stage in women’s lives to help alleviate postmenopausal health problems.
